# *Asparagopsis taxiformis* as a Novel Antioxidant Ingredient for Climate-Smart Aquaculture: Antioxidant, Metabolic and Digestive Modulation in Juvenile White Seabream (*Diplodus sargus*) Exposed to a Marine Heatwave

**DOI:** 10.3390/antiox13080949

**Published:** 2024-08-05

**Authors:** Alícia Pereira, Isa Marmelo, Marta Dias, Ana Catarina Silva, Ana Catarina Grade, Marisa Barata, Pedro Pousão-Ferreira, Jorge Dias, Patrícia Anacleto, António Marques, Mário S. Diniz, Ana Luísa Maulvault

**Affiliations:** 1 IPMA—Portuguese Institute for the Sea and Atmosphere, Avenida Alfredo Magalhães Ramalho 6, 1495-165 Algés, Portugal; isa.marmelo@ipma.pt (I.M.); ana.catarina.silva@ipma.pt (A.C.S.); agrade@ipma.pt (A.C.G.); mbarata@ipma.pt (M.B.); pedro.pousao@ipma.pt (P.P.-F.); panacleto@ipma.pt (P.A.); amarques@ipma.pt (A.M.); aluisa@ipma.pt (A.L.M.); 2 UCIBIO REQUIMTE, Applied Molecular Biosciences Unit, NOVA School of Science and Technology, NOVA University of Lisbon, 2829-516 Caparica, Portugal; maddias@fc.ul.pt (M.D.); mesd@fct.unl.pt (M.S.D.); 3 CIIMAR, Interdisciplinary Centre of Marine and Environmental Research, University of Porto, Porto, Terminal de Cruzeiros do Porto de Leixões, Avenida General Norton de Matos s/n, 4450-208 Matosinhos, Portugal; 4 MARE, Marine and Environmental Sciences Centre & ARNET, Aquatic Research Infrastructure Network Associate Laboratory, Faculty of Sciences, University of Lisbon (FCUL), 1749-016 Lisbon, Portugal; 5 Associate Laboratory i4HB, Institute for Health and Bioeconomy, NOVA School of Science and Technology, NOVA University of Lisbon, 2829-516 Caparica, Portugal; 6 SPAROS Lda., Área Empresarial de Marim, Lote C, 8700-221 Olhão, Portugal; jorgedias@sparos.pt

**Keywords:** antioxidant response, metabolic response, digestive enzymes, histopathology, climate change, farmed fish, macroalgae

## Abstract

The increasing frequency and duration of marine heatwaves (MHWs) due to climate change pose severe threats to aquaculture, causing drastic physiological and growth impairments in farmed fish, undermining their resilience against additional environmental pressures. To ensure sustainable production that meets the global seafood demand and animal welfare standards, cost-effective and eco-friendly strategies are urgently needed. This study explored the efficacy of the red macroalga *Asparagopsis taxiformis* on juvenile white seabream *Diplodus sargus* reared under optimal conditions and upon exposure to a MHW. Fish were fed with four experimental diets (0%, 1.5%, 3% or 6% of dried powdered *A. taxiformis*) for a prophylactic period of 30 days (T30) and subsequently exposed to a Mediterranean category II MHW for 15 days (T53). Biometric data and samples were collected at T30, T53 and T61 (8 days post-MHW recovery), to assess performance indicators, biomarker responses and histopathological alterations. Results showed that *A. taxiformis* supplementation improved catalase and glutathione S-transferase activities and reduced lipid peroxidation promoted by the MHW, particularly in fish biofortified with 1.5% inclusion level. No histopathological alterations were observed after 30 days. Additionally, fish biofortified with 1.5% *A. taxiformis* exhibited increased citrate synthase activity and fish supplemented with 1.5% and 3% showed improved digestive enzyme activities (e.g., pepsin and trypsin activities). Overall, the present findings pointed to 1.5% inclusion as the optimal dosage for aquafeeds biofortification with *A. taxiformis*, and confirmed that this seaweed species is a promising cost-effective ingredient with functional properties and great potential for usage in a climate-smart context.

## 1. Introduction

Climate change effects are expected to have severe consequences in the aquaculture sector [1] due to significant shifts in temperature and occurrence of extreme weather events, such as marine heatwaves (MHWs) [2]. MHWs are events of unusually elevated temperatures persisting for a minimum of five consecutive days and extending to several months, caused by a combination of oceanographic and atmospheric processes [3]. The frequency and duration of these extreme events have risen by 54% over the past century [4], and climate projections indicate that their occurrence will continue to increase in the future, both in terms of intensity and frequency [5,6]. Outdoor aquaculture facilities are extremely vulnerable to environmental variations, especially when they take place in sudden and extreme ways, such as during MHWs [7,8,9]. Indeed, abrupt fluctuations in seawater temperature can impact the physiological and metabolic responses of farmed fish species (and early life stages, in particular), resulting in compromised growth, performance and, ultimately, survival [9,10,11]. This, consequently, affects their resilience against exposure to additional environmental stressors [12]. The impacts that climate change-related stressors can have upon farmed species have, thus, recently become a major focus of attention for aquaculture researchers and producers, as they can have serious animal welfare and economic repercussions [2,13,14]. Although research in this direction is still rather limited, especially in what concerns the effects prompted by extreme weather events, such as MHWs [15], a few recent studies have reported higher lipid peroxidation, a shift in metabolic pathways and increased expression of heat shock protein (HSP70) in different fish species exposed to these conditions [11,16,17].

Apart from identifying threats and impacts to aquaculture’s sustainable growth, it is also imperative to develop climate-smart strategies that improve the resilience of this sector and assure sufficient, sustainable and high-quality production of farmed seafood, even under adverse rearing conditions. In this sense, adaptation strategies based on nutritional interventions (i.e., development of tailor-made eco-innovative aquafeeds formulated with novel functional ingredients) are currently considered a key management strategy in fish farming to ameliorate the adverse impacts of thermal stress [18,19,20,21]. Dietary manipulation is typically appealing to the industry due to the easiness of implementation and absence of adverse effects on the environment or human health [22]. The primary commercial factor for incorporating new feed ingredients is the potential influence on farmed fish production, for which improvements in growth, fitness or nutrient conversion efficiency directly yield economic benefits to farmers [23]. However, in order to successfully achieve these goals, it is crucial to assure that the ingredients in aquafeeds are: (i) nutritious, non-harmful and non-repulsive (in terms of taste and odor) to farmed species; (ii) easily acquired or sustainably produced in amounts that are compatible with the aquaculture industrial scale; and (iii) affordable, not substantially increasing the costs associated with feeds/fish production and, ultimately, products’ final price to consumers.

Seaweeds’ outstanding potential as a functional ingredient for incorporation in aquafeed formulations has recently received great attention in research devoted to aquaculture nutrition, as these species exhibit a rich biodiversity and are abundant in the natural environment [24,25]. Moreover, they are known to be rich in bioactive compounds with beneficial properties to both animals and humans (e.g., antioxidant, anti-inflammatory, antibacterial), including polysaccharides (alginates, agar, β-glucan, mannitol and carrageenans), polyphenols (phenolic acids, phlorotannins, flavonoids and halogenated derivatives), and carotenoids (fucoxanthin and β-carotene) [26,27]. These compounds can be converted into various secondary metabolites with diverse biological activities, contributing to improved growth performance, enhanced physiological activity, bolstered immune defense, increased antioxidant capacity and a more effective stress response [13,28,29]. Employing natural ingredients like macroalgae in fish diets is a more environmentally conscious option compared to incorporating synthetic ingredients. Additionally, this approach is less likely to encounter resistance from consumers to the consumption of farmed fish [30,31]. A seaweed species attracting considerable interest due to its abundant natural product chemistry and bioactive properties is the red marine macroalga *Asparagopsis taxiformis*. Native to the warm temperature waters of the Indo-Pacific, this species is currently undergoing commercial farming development in Europe [23,32]. In addition, it exhibits a strong invasive behavior and is considered a pest in European waters [33,34]; therefore, it has potential for wild harvesting with minimal environmental consequences [23]. Recent studies have also confirmed that this seaweed can, indeed, improve farmed fish resilience and immunity [23]. Still, the assets observed upon biofortification with *A. taxiformis* are limited to optimal growth conditions, as no study so far has investigated the efficiency of this eco-innovative strategy in environmentally stressful contexts such as those elicited by climate change-related stressors. Addressing this knowledge gap is utmost important for the sustainable and profitable expansion of aquaculture in the face of the prevailing environmental conditions of tomorrow’s oceans.

The white seabream (*Diplodus sargus*), commonly found in Southern Europe, is an important seafood species for fisheries and aquaculture, being highly appreciated among European consumers due to its organoleptic properties, quality flesh and favorable price [35,36]. Recent findings indicate that seabreams are susceptible to the impacts of climate change, given their proximity to physiological thresholds and limited capacity of acclimation plasticity to changing conditions [37,38]. Considering the current lack of empirical studies in this direction, the assessment of fish growth performance, metabolic status and antioxidant enzymes may, certainly, provide crucial clues that will help in understanding the resilience of farmed *D. sargus* [9,12]. In addition, in order to validate the use of a given experimental functional aquafeed, it is also essential to study the digestive functionality and efficiency in nutrient conversion, as the dietary habits and digestive strategies strongly vary among fish species [39,40,41].

Within this context, the aim of the present study was to investigate the effectiveness of aquafeeds biofortified with the red macroalga *A. taxiformis*, at three different inclusion levels (1.5%, 3% and 6%), in enhancing the antioxidant, metabolic and digestive responses of juvenile white seabream *D. sargus* upon exposure to a MHW equivalent to category II in the Mediterranean region.

## 2. Materials and Methods

### 2.1. Experimental Diets

Four experimental functional diets (2.0 mm pellets) were formulated to be isonitrogenous (46.9% crude protein), isolipidic (16.1% crude fat) and isoenergetic (20.0 MJ kg^−1^) by SPAROS Lda. (Olhão, Portugal). The commercial control diet (CTR) used in this study did not include any macroalga supplementation and was formulated according to the nutritional requirements of juvenile *D. sargus*. Based on the control diet formulation, three additional experimental diets were biofortified with dried powdered *A. taxiformis*, at three specific inclusion percentages: 1.5% (1.5-AT; low, representing the most economically feasible option), 3% (3-AT; average) and 6% (6-AT; high, albeit less economically viable), replacing wheat meal (Table 1). The red macroalga was collected in the gametophyte life stage by the seaExpert company at Angústias dock, Fail Island, Azores, Portugal. Subsequently, the seaweed was dried using a Black Block solar dryer (BBKW, Lisboa, Portugal) under dark conditions at 40 °C for a duration of 2 days.

### 2.2. Feeding Trial and Fish Sampling

Juvenile white seabream (*D. sargus*) specimens (28.50 ± 1.10 g total weight; 12.2 ± 0.3 cm total length; mean ± standard deviation, *n* = 210) were obtained from the Aquaculture Research Station of the Portuguese Institute for the Sea and Atmosphere (EPPO-IPMA, Olhão, Portugal) and maintained for 3 weeks in a quarantine system (composed of two tanks with 660 L total capacity each) at IPMA’s Live Marine Organisms Bioterium (LABVIVOS, Algés, Portugal) before entering the experimental setup. Throughout the quarantine period, fish were kept under the following conditions: (i) temperature: 24.0 ± 0.5 °C; (ii) dissolved oxygen: 7.2 ± 0.2 mg L^−1^; (iii) salinity: 35.0 ± 0.5‰; (iv) pH: 8.0 ± 0.1; and (v) photoperiod: 12 h light/12 h dark. They were hand-fed twice a day with the CTR feed, equivalent to 2% of their average body weight (BW).

**Figure 1:**
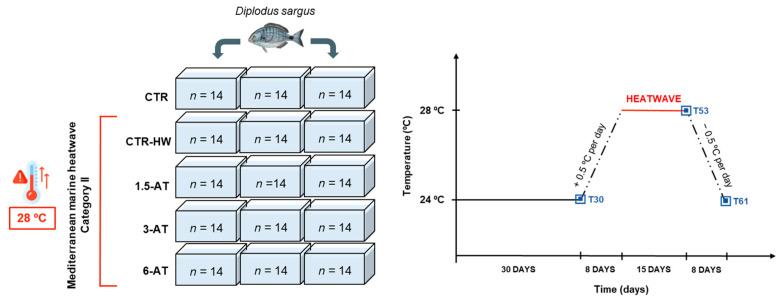
Experimental design and simulation of the category II Mediterranean Marine Heatwave including sampling days (T30, T53 and T61) for white seabream, *D. sargus*, fed with the different diets. Days 1–30, biofortification at 24 °C; day 30, first sampling (T30); days 30–38, temperature ramp to 28 °C; days 38–53, category II marine heatwave (28 °C); day 53, sampling day after exposure to peak temperature of the MHW (T53); days 53–61, temperature ramp back to 24 °C; day 61, sampling day post-MHW (T61). Abbreviations: CTR—control feed; CTR-HW—control feed and exposure to the MHW; 1.5-AT—1.5% inclusion of *A. taxiformis*; 3-AT—3% inclusion of *A. taxiformis*; 6-AT—6% inclusion of *A. taxiformis*.

After the quarantine period, fish were equally and randomly allocated to 15 rectangular glass tanks, each with a total volume of 500 L, within the experimental recirculation aquaculture systems (RAS). Each RAS was equipped with a physical filter [composed of a filter bag (400 micron; TMC Iberia, Loures, Portugal); filter sponge and glass wool], a biological filter (Bio Balls 1.5” Aquarium Pond Filter, TMC Iberia, Loures, Portugal), a UV sterilizer (ClearUVC-36, EHEIM, Deizisau, Germany), a protein skimmer (Tornado 120, Mantis) and submerged air stones to control dissolved oxygen. Additionally, tanks included submerged digital thermostats (300 W, V^2^Therm Digital Heaters, TMC Iberia, Loures, Portugal) along with an automatic seawater refrigeration system (Foshan Weinuo Refrigeration Equipment Co., Ltd., Foshan, China), both integrated into a computerized aquarium control system (ProfiLux 3 Outdoor, GHL, Kaiserslautern, Germany), for temperature regulation in each tank. Five treatments were carried out, each of them in triplicate (i.e., 3 tanks/treatment = 15 tanks total, *n* = 14 fish per replicate tank, 42 per treatment in total; Figure 1): (i) CTR (control treatment)—animals fed with CTR feed (without macroalga supplementation); (ii) CTR-HW (control + heatwave)—animals fed with CTR feed and exposed to a marine heatwave (MHW); (iii) 1.5-AT—animals fed with aquafeed biofortified with 1.5% of *A. taxiformis* and exposed to a MHW; (iv) 3-AT—animals fed with aquafeed biofortified with 3% of *A. taxiformis* and exposed to a MHW; and (v) 6-AT—animals fed with aquafeed biofortified with 6% of *A. taxiformis* and exposed to a MHW. Following a seven-day acclimation period to the experimental setup, fish from each treatment were fed (2% BW) with the respective diet for 30 consecutive days (biofortification period) while being kept at 24 °C (corresponding to the average summer temperature found in Mediterranean coastal areas where semi-intensive aquaculture facilities are settled; [42]). Summer seawater temperature was set as the reference value, as it is during this season that MHWs strike the most and in more intense ways (i.e., worst-case scenario). Additionally, these temperature values are in line with the annual trends registered in Ria Formosa (Olhão; data from Portuguese Institute for the Sea and Atmosphere—IPMA, I.P.), where juvenile *D. sargus* were reared until transplantation to laboratorial conditions.

After this period, a category II (strong) MHW typical of the Mediterranean area [43] was simulated by gradually increasing seawater temperature over 8 days (+0.5 °C per day, i.e., 8 days of temperature ramp increase; final ΔTemperature = +4 °C) in all treatments (except for CTR treatment, which remained at 24 °C throughout the entire experimental trial), followed by a “plateau” period of 15 days at the peak temperature (i.e., 28 °C). Subsequently, a recovery period was undertaken by slowly decreasing the temperature until reaching 24 °C again in all treatments (−0.5 °C per day, i.e., 8 days of temperature ramp decrease; ΔTemperature = −4 °C; Figure 1). During the trial, the remaining abiotic parameters (apart from temperature) were maintained as follows: dissolved oxygen at 7.2 ± 0.2 mg L^−1^, salinity at 35.0 ± 0.5‰, pH at 8.0 ± 0.1 and a photoperiod of 12 h light/12 h dark regime. Temperature was checked with a portable precision thermometer (TFX 430, Ebro Electronic, Germany), while salinity, pH and dissolved oxygen were monitored daily using a multi-parameter measuring instrument (Multi 3420 SET G, WTW, Weilheim, Germany). Adjustments were made whenever necessary to ensure all parameters remained within the set values. Ammonia (NH_3_/NH_4_), nitrites (NO_2_^−^), and nitrates (NO_3_^−^) levels were checked daily using colorimetric tests (Salifert, Duiven, The Netherlands) and were maintained within optimal levels, with nitrates kept below 50 mg L^−1^. Fish faeces were removed daily along with 25% of seawater renewal in each tank. No mortality was recorded throughout the trial.

On days 30 (T30, i.e., after 30 days of biofortification), 53 (T53, i.e., after 15 days of exposure to the MHW peak temperature) and 61 (T61, i.e., after 8 days of recovery from the MHW) of the feeding trials, 12 fish were randomly selected from each treatment (4 fish collected from each of the 3 replicate tanks per treatment) and euthanized by immersion in tricaine methanesulfonate solution at 2000 mg L^−1^ (MS-222, Acros Organics, Geel, Belgium) buffered with sodium bicarbonate (NaHCO_3_, Sigma-Aldrich, St. Louis, MI, USA) for 10 min. Fish were fasted 24 h prior to each sampling event to prevent contamination of samples with faeces and undigested feed content in the digestive tract. All fish were measured (total length, TL), weighed (W) and samples of muscle, gut and liver tissues were collected (approximately 100 mg of each tissue). Liver weight was registered for subsequent hepatosomatic index (HSI) determination. Fish tissues (*n* = 6) were homogenized in different buffer solutions according to the methodological requirements of antioxidant, metabolic and digestive biomarkers (see Section 2.4). Tissue homogenates were frozen and stored at −80 °C until biochemical biomarker analyses were conducted. Gut and liver samples (*n* = 3) were also taken for histopathological analysis.

### 2.3. Fish Growth and Feed Efficiency

Fish performance and body condition indexes were calculated based on BW (body weight) and body total length (TL), using the following formula:

Condition factor (K) = BW (g)/TL^3^ (cm) × 100 (1)

Hepatosomatic index (HSI, %) = liver weight (g)/BW (g) × 100(2)

Weight gain (GW, %) = (final BW (g) − initial BW (g))/initial BW (g) × 100(3)

Specific growth rate (SGR, % day^−1^) = [Ln(final BW) − Ln (initial BW)]/days × 100 (4)

Feed efficiency was determined using feed conversion ratio (FCR), as follows:

FCR = dry feed supplied/fish wet weight gained(5)

### 2.4. Biochemical Analyses

Enzyme activities were assessed using well-established protocols previously optimized for fish tissues [44]. All assays were modified for 96-well microplates and conducted with a microplate reader (Thermo Scientific Multiskan GO 1510, Waltham, MA, USA). Each sample was analyzed at least in triplicate, using reagents and standards of *pro analysis* grade or higher quality. Biomarker results for all samples were normalized based on the respective total protein content (expressed in mg of protein; except for SOD, % inhibition), determined according to the Bradford assay [45].

#### 2.4.1. Oxidative Stress

Fish muscle, gut and liver tissues were homogenized in ice-cold conditions with 1 mL of phosphate-buffered saline pH 7.4 (PBS, 0.14 M NaCl, 0.003 M KCl, 0.01 M Na_2_HPO_4_, 0.002 M KH_2_PO_4_; reagents from Sigma-Aldrich, Taufkirchen, Bavaria, Germany) using an Ultra-Turrax device (T10 basic, Ika, Staufen, Baden-Württemberg, Germany). Crude homogenates were centrifuged in 1.5 mL microtubes at 10,000× *g* and 4 °C for 10 min and the supernatants were transferred to new microtubes and frozen (−80 °C) until further analyses. Supernatants were used to determine the following oxidative stress biomarkers: catalase (CAT) activity, glutathione S-transferase (GST) activity, superoxide dismutase (SOD) activity and lipid peroxidation (LPO). The methods are described in detail in Marmelo et al. [46].

#### 2.4.2. Metabolic Enzymes

Citrate synthase (CS) and lactate dehydrogenase (LDH) activities were assessed in the muscle of juvenile *D. sargus*, using the procedures outlined in Rosa et al. [47]. The samples were homogenized under ice-cold conditions with 1 mL of phosphate-buffered saline solution pH 7.4 tailored for each analysis: (a) CS—20 mM HEPES (Gibco, Grand Island, NY, USA), 1 mM EDTA (Triplex, Merk, Geneva, Switzerland), Triton 1% (Sigma Aldrich, Taufkirchen, Bavaria Germany); and (b) LDH—150 mM Imidazole (Carl Roth, Karlsruhe, Baden-Württemberg, Germany), 1 mM EDTA (Triplex), Triton 1%. After homogenization, homogenates were centrifuged in 1.5 mL microtubes at 10,000× *g* for 10 min (4 °C), and the resulting supernatant was transferred to new microtubes and stored at −80 °C until further analyses.

#### 2.4.3. Digestive Enzymes

To determine digestive enzyme activity, the entire digestive tract of white seabream was homogenized in 1 mL of homogenization buffer for digestive enzymes (1 mM Tris-HCl, 0.1 mM EDTA, 0.1% Triton, pH 7.8). Crude homogenates were centrifuged in 1.5 mL microtubes at 14,000× *g* for 30 min at 4 °C and the resulting supernatant was transferred to new microtubes and frozen at −80 °C until further analyses.

Amylase activity was determined using the method described in Zaharudin et al. [48] and starch as substrate. Pepsin activity was determined according to the methods described by Anson [49], Worthington [50], and Comabella et al. [51], using haemoglobin 2.5% at pH 2 as substrate. Trypsin activity was determined based on the procedures described by Erlanger et al. [52] and Klomklao et al. [53], using BAPNA (Nα-benzoyl-DL-arginine 4-nitroanilide; Sigma Aldrich, Taufkirchen, Bavaria, Germany) as substrate.

### 2.5. Histological Analysis

The livers and intestines were fixed in 10% formalin phosphate-buffered solution for 24 h at room temperature and then stored in 75% ethanol until further use. Subsequently, the tissues were placed in cassettes and processed in the tissue processor (Leica, TP 1020), where they were dehydrated using increasing concentrations of ethanol (70%, 90%, 96% and absolute), xylene, paraffin + xylene, paraffin 42–44 °C and paraffin 56–58 °C. The tissues were embedded in paraffin (MERCK, melting point 56–58 °C) using a modular tissue embedding center (Leica, EG1150). The tissues were then cooled and sectioned (3–4 µm) using a microtome (Leica RM2125 RTS). The samples were stained using a slide staining processor (Leica Autostainer XL, ST5010) with haematoxylin and eosin (H&E) stain. The slides were then mounted with Entellan™ (1079610) and allowed to dry for 24 h. Finally, samples were examined under an optical microscope (ZEISS, 451889 Axioplan 2) coupled to a microscope digital video camera (Leica MC190 HD).

### 2.6. Data Analysis

To assess significant differences between treatments in animal fitness indexes, feed efficiency and biomarker levels, one-way nested ANOVA analyses were employed, using the sampling time (T30, T53 and T61) as nesting factor. Prior to analysis, normality (Kolmogorov-Smirnov’s test) and homogeneity of variances (Levene’s test) were evaluated. Data were square-rooted whenever one of the ANOVA assumptions was not verified. Post-hoc Tukey HSD tests were conducted for multiple comparisons when significant differences were identified at a significance level of 0.05. STATISTICATM software (Version 7.0, StatSoft Inc., Tulsa, OK, USA) was used for all statistical analyses.

## 3. Results

### 3.1. Fish Growth and Feed Efficiency

Growth performance and fitness indexes are presented in Table 2. Weight (W), total length (TL) and hepatosomatic index (HSI) were not significantly affected by treatment or sampling day (*p* > 0.05).

Weight gain (WG) showed no significant differences between treatments on days 30 and 53 (*p* > 0.05). However, WG was 30% lower in CTR-HW treatment compared to the CTR treatment on day 61 (*p* < 0.05). Additionally, fish fed with 6% *A. taxiformis* exhibited significantly lower WG compared to fish from the CTR-HW treatment on T61 (corresponding to a 30% decrease; *p* < 0.05).

### 3.2. Oxidative Stress

**Figure 2:**
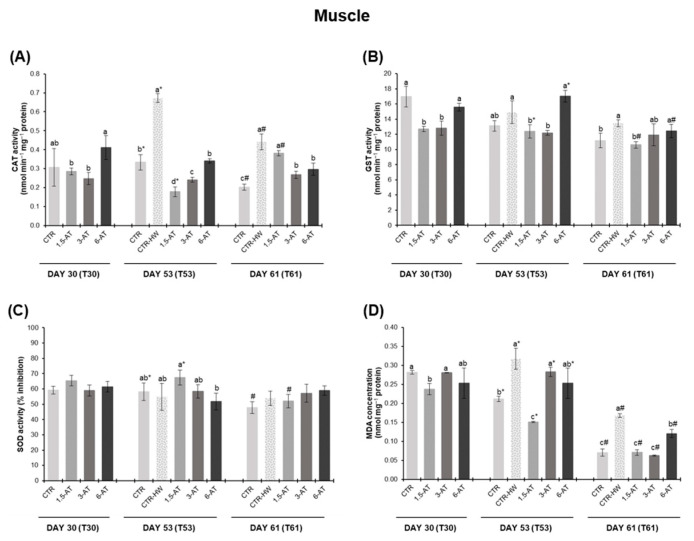
Oxidative stress biomarkers in the muscle tissue of white seabream, *D. sargus*, juveniles fed with the different diets at days 30 (T30, i.e., after 30 days of biofortification), 53 (T53, i.e., after 15 days of exposure to the MHW peak temperature), and 61 (T61, i.e., after 8 days of recovery from the MHW) of the trial (mean ± SD, *n* = 6). (**A**)—catalase (CAT) activity (nmol min^−1^ mg^−1^ protein); (**B**)—glutathione S-transferase (GST) activity (nmol min^−1^ mg^−1^ protein); (**C**)—superoxide dismutase (SOD) activity (% inhibition); and (**D**)—lipid peroxidation (LPO, expressed as MDA concentration, nmol mg^−1^ protein). Different letters denote significant differences between treatments on the same sampling day, and different symbols (* and #) indicate significant differences between sampling days T53 and T61 for the same treatment (*p* < 0.05). The absence of letters or symbols indicates no statistical difference. Abbreviations: CTR—control feed; CTR-HW—control feed exposed to the MHW; 1.5-AT—feed with 1.5% inclusion of *A. taxiformis*; 3-AT—feed with 3% inclusion of *A. taxiformis*; 6-AT—feed with 6% inclusion of *A. taxiformis*; CAT—catalase; GST—glutathione S-transferase; SOD—superoxide dismutase; MDA—malondialdehyde.

Figure 2 presents fish antioxidant enzyme activities (CAT, GST and SOD) and lipid peroxidation (LPO) in muscle tissue of white seabream juveniles. On day 30, prior to the onset of the MHW, muscle CAT and SOD activities were not significantly affected by the supplementation of *A. taxiformis* in fish diets (*p* > 0.05; Figure 2A,C). GST activity was significantly lower in fish fed with 1.5% and 3% *A. taxiformis* in relation to the CTR treatment (*p* < 0.05; Figure 2B). Concerning LPO, only fish fed with 1.5% macroalga exhibited lower LPO values than the CTR treatment (*p* < 0.05; Figure 2D). On day 53, CAT activity was significantly higher in the CTR-HW treatment compared to the CTR treatment (*p* < 0.05). When comparing the treatments exposed to the MHW, fish biofortified with *A. taxiformis* revealed significantly lower CAT activity than non-biofortified ones (i.e., CTR-HW; *p* < 0.001), with 1.5-AT treatment showing the lowest activity (corresponding to a 73.5% reduction; Figure 2A). Regarding GST, only fish fed with 1.5% and 3% *A. taxiformis* showed a significant decrease in relation to the CTR-HW treatment (*p* < 0.05; Figure 2B). SOD activity did not significantly differ between treatments (*p* > 0.05; Figure 2C). LPO levels were significantly higher in fish from the CTR-HW in relation to the CTR treatment (*p* < 0.001). Regardless of the changes observed in antioxidant enzyme activities, only fish fed with 1.5% *A. taxiformis* exhibited significantly lower LPO values than those in the CTR-HW treatment (*p* < 0.001; Figure 2D). By day 61 (recovery from MHW), CAT and GST activities were still significantly higher in the CTR-HW treatment compared to the CTR treatment (*p* < 0.001 and *p* < 0.05, respectively) as well as LPO levels (*p* < 0.001). Additionally, fish fed with 3% and 6% macroalga also displayed significantly reduced CAT activity levels in comparison to the CTR-HW treatment (*p* < 0.05; Figure 2A). Among the macroalga supplementation treatments, only fish fed with 1.5% *A. taxiformis* showed significantly lower GST activity than those from the CTR-HW treatment (*p* < 0.05; Figure 2B). SOD activity levels remained similar across all treatments upon recovery from the MHW (*p* > 0.05). Biofortified fish exhibited a significant decrease in LPO levels compared to non-biofortified fish exposed to MHW, i.e., a 58%, 63% and 29% reduction in 1.5-AT, 3-AT and 6-AT treatments, respectively, in relation to CTR-HW treatment (*p* < 0.05; Figure 2D).

Comparing results after 15 days of exposure to MHW peak temperature (day 53) with those upon 8 days of recovery from MHW (day 61) within the same treatment, CAT activity showed a significant decrease of 34% in the CTR-HW treatment (*p* < 0.001), while 1.5-AT treatment showed a significant increase of 53% (*p* < 0.001; Figure 2A). GST activity on day 61 differed significantly from day 53 only in the 1.5% and 6% *A. taxiformis* treatments, where a reduction of 14% and 27% in activity levels was observed, respectively (*p* < 0.05; Figure 2B). In terms of SOD activity, only the 1.5-AT treatment differed statistically from day 53 (*p* < 0.05; Figure 2C). Lastly, LPO significantly decreased in all treatments during the recovery from the MHW (i.e., day 61; *p* < 0.05), with the 3-AT treatment showing the most significant reduction, equivalent to a 60% decrease (*p* < 0.001; Figure 2D).

**Figure 3:**
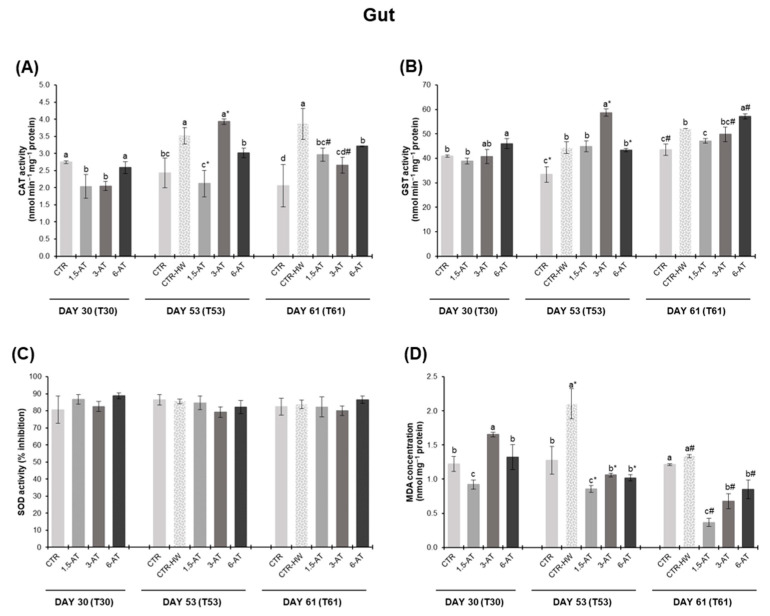
Oxidative stress biomarkers in the gut tissue of white seabream, *D. sargus*, juveniles fed with the different diets at days 30 (T30, i.e., after 30 days of biofortification), 53 (T53, i.e., after 15 days of exposure to the MHW peak temperature), and 61 (T61, i.e., after 8 days of recovery from the MHW) of the trial (mean ± SD, *n* = 6). (**A**)—catalase (CAT) activity (nmol min^−1^ mg^−1^ protein); (**B**)—glutathione S-transferase (GST) activity (nmol min^−1^ mg^−1^ protein); (**C**)—superoxide dismutase (SOD) activity (% inhibition); and (**D**)—lipid peroxidation (LPO, expressed as MDA concentration, nmol mg^−1^ protein). Different letters denote significant differences between treatments on the same sampling day, and different symbols (* and #) indicate significant differences between sampling days T53 and T61 for the same treatment (*p* < 0.05). The absence of letters or symbols indicates no statistical difference. Abbreviations: CTR—control feed; CTR-HW—control feed exposed to the MHW; 1.5-AT—feed with 1.5% inclusion of *A. taxiformis*; 3-AT—feed with 3% inclusion of *A. taxiformis*; 6-AT—feed with 6% inclusion of *A. taxiformis*; CAT—catalase; GST—glutathione S-transferase; SOD—superoxide dismutase; MDA—malondialdehyde.

Oxidative stress biomarkers in fish gut are presented in Figure 3. At day 30 of the trial, CAT activity showed a significant decrease in fish fed with 1.5% and 3% *A. taxiformis* compared to those in the CTR treatment (*p* < 0.05; Figure 3A). As for gut GST, fish from the 6-AT treatment exhibited significantly higher activity levels than those in the CTR treatment (*p* < 0.05; Figure 3B). No significant differences were observed among treatments in terms of SOD activity (*p* > 0.05; Figure 3C). LPO content was significantly lower in fish fed with 1.5% macroalga compared to the CTR treatment (*p* < 0.05), while fish from the 3-AT treatment revealed a significant increase (*p* < 0.05; Figure 3D). Regarding the effect of the MHW (day 53), significantly higher CAT and GST activities were observed in CTR-HW compared to the CTR treatment (*p* < 0.05). Additionally, CAT values were significantly lower in fish fed the 1.5-AT and 6-AT diets in relation to CTR-HW, representing a 40% and 14% reduction, respectively (*p* < 0.05; Figure 3A). GST activity was induced in fish fed with 3% *A. taxiformis* compared to CTR-HW (*p* < 0.05; Figure 3B). In comparison with the CTR treatment, significantly elevated LPO contents were observed in the CTR-HW (*p* < 0.001). Furthermore, LPO levels were decreased in biofortified fish (compared to CTR-HW), with the lowest recorded values being observed in fish fed the 1.5-AT diet (*p* < 0.001; Figure 3D).

After the MHW recovery period (day 61), higher levels of CAT and GST activity were observed in the CTR-HW treatment compared to the CTR (*p* < 0.001). All diets supplemented with *A. taxiformis* exhibited a decrease in CAT activity compared to CTR-HW (*p* < 0.05; Figure 3A). In terms of GST, fish fed 1.5% macroalga showed a significant reduction in activity, while those fed with 6% revealed higher activity levels (*p* < 0.05; Figure 3B). No significant differences were found between the CTR and CTR-HW treatments and the macroalga-biofortified treatments regarding SOD activity (*p* > 0.05). As for LPO, significantly lower values were found in biofortified fish (regardless of seaweed inclusion level) compared to CTR-HW treatment, with the lowest levels recorded in the 1.5% *A. taxiformis* treatment (equivalent to a 73% reduction; *p* < 0.001; Figure 3D).

Regarding the recovery period (i.e., between T53 and T61), only the 3-AT treatment showed a significant decrease of 32% and 15% in CAT and GST activities, respectively (*p* < 0.001). Conversely, 1.5-AT and 6-AT treatments revealed a significant increase of 28% and 24% in CAT and GST activity levels, respectively (*p* < 0.05; Figure 3A,B). MDA concentration significantly decreased in all treatments upon recovery from the MHW, regardless of biofortification (*p* < 0.05), with the 1.5-AT treatment showing the most significant reduction, equivalent to a 57% decrease (*p* < 0.001; Figure 3D).

**Figure 4:**
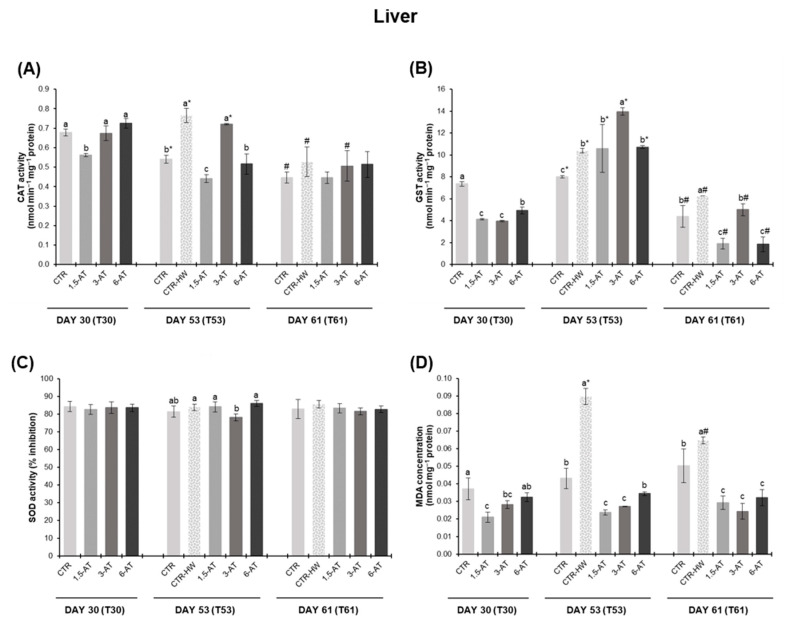
Oxidative stress biomarkers in the liver of white seabream, *D. sargus*, juveniles fed with the different diets at days 30 (T30, i.e., after 30 days of biofortification), 53 (T53, i.e., after 15 days of exposure to the MHW peak temperature), and 61 (T61, i.e., after 8 days of recovery from the MHW) of the trial (mean ± SD, *n* = 6). (**A**)—catalase (CAT) activity (nmol min^−1^ mg^−1^ protein); (**B**)—glutathione S-transferase (GST) activity (nmol min^−1^ mg^−1^ protein); (**C**)—superoxide dismutase (SOD) activity (% inhibition); and (**D**)—lipid peroxidation (LPO, expressed as MDA concentration, nmol mg^−1^ protein). Different letters denote significant differences between treatments on the same sampling day, and different symbols (* and #) indicate significant differences between sampling days T53 and T61 for the same treatment (*p* < 0.05). The absence of letters or symbols indicates no statistical difference. Abbreviations: CTR—control feed; CTR-HW—control feed exposed to the MHW; 1.5-AT—feed with 1.5% inclusion of *A. taxiformis*; 3-AT—feed with 3% inclusion of *A. taxiformis*; 6-AT—feed with 6% inclusion of *A. taxiformis*; CAT—catalase; GST—glutathione S-transferase; SOD—superoxide dismutase; MDA—malondialdehyde.

Antioxidant responses in fish liver are presented in Figure 4. Starting with CAT activity, after 30 days of biofortification, only fish fed with 1.5% *A. taxiformis* significantly differed from those in the CTR treatment (*p* < 0.05; Figure 4A), while GST and LPO were significantly lower in all biofortified treatments, with the lowest values being recorded in the 1.5-AT and 3-AT treatments (*p* < 0.001; Figure 4B,D). On day 53, higher levels of liver CAT and GST activity were observed in the CTR-HW treatment compared to the CTR (*p* < 0.001 and *p* < 0.05, respectively). In the case of CAT, a significant decrease corresponding to 42% and 32% was observed in 1.5-AT and 6-AT, respectively, compared to the CTR-HW (*p* < 0.001; Figure 4A). Fish fed with 3% *A. taxiformis* revealed induced GST activity (equivalent to a 34% increase compared to CTR-HW; *p* < 0.001; Figure 4B). Conversely, SOD inhibition was significantly lower in fish fed the 3-AT diet (*p* < 0.05; Figure 4C). LPO values in the CTR-HW treatment were significantly higher than those in the CTR (*p* < 0.001). Additionally, all supplemented diets displayed significantly lower LPO values compared to CTR-HW, with lower levels observed in the 1.5-AT and 3-AT treatments (*p* < 0.001; Figure 4D). At day 61, GST activity and lipid peroxidation revealed higher values in CTR-HW treatment compared to CTR (*p* < 0.05; Figure 4B,D). All *A. taxiformis* supplemented-treatments exhibited significantly decreased GST activity levels and LPO contents compared to CTR-HW, with the lowest values observed in the 1.5-AT and 6-AT treatments in terms of GST activity (*p* < 0.001; Figure 4B).

Concerning the recovery period, fish from the CTR-HW and 3-AT treatments exhibited reduced levels of CAT activity at day 61 compared to day 53 (equivalent to a 31% and 30% reduction, respectively; *p* < 0.001; Figure 4A). In the case of GST, all treatments revealed decreased activity levels at day 61 (*p* < 0.001), with the 6-AT treatment showing the most significant reduction equivalent to an 83% decrease (*p* < 0.001; Figure 4B). Lastly, only fish from the CTR-HW treatment displayed decreased levels of LPO at day 61 (28% decrease in relation to day 53; *p* < 0.001; Figure 4D).

### 3.3. Metabolic Responses

**Figure 5:**
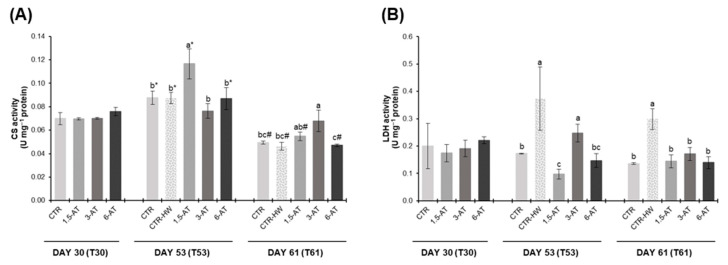
Metabolic responses in the muscle of *D. sargus* juveniles fed with the different diets at days 30 (T30, i.e., after 30 days of biofortification), 53 (T53, i.e., after 15 days of exposure to the MHW peak temperature), and 61 (T61, i.e., after 8 days of recovery from the MHW) of the trial (mean ± SD, *n* = 6). (**A**)—citrate synthase (CS) activity (U mg^−1^ protein) and (**B**)—lactate dehydrogenase (LDH) activity (U mg^−1^ protein). Different letters denote significant differences between treatments on the same sampling day, and different symbols (* and #) indicate significant differences between sampling days T53 and T61 for the same treatment (*p* < 0.05). The absence of letters or symbols indicates no statistical difference. Abbreviations: CTR—control feed; CTR-HW—control feed exposed to the MHW; 1.5-AT—feed with 1.5% inclusion of *A. taxiformis*; 3-AT—feed with 3% inclusion of *A. taxiformis*; 6-AT—feed with 6% inclusion of *A. taxiformis*; CS—citrate synthase; LDH—lactate dehydrogenase.

Fish muscle metabolic responses are presented in Figure 5. Upon 30 days of biofortification with *A. taxiformis*, CS and LDH activities remained unchanged across treatments (*p* > 0.05). On day 53 (exposure to MHW), higher LDH activity was observed in CTR-HW treatment compared to the CTR (*p* < 0.001), but no significant differences were found in CS activity (*p* > 0.05). A significant increase of 33% was observed in CS levels of fish fed with 1.5% *A. taxiformis* in relation to the CTR-HW treatment (*p* < 0.001; Figure 5A). Additionally, decreased LDH activity was also found in fish from the 1.5-AT and 6-AT treatments (*p* < 0.001; Figure 5B). Lastly, by day 61 of the trial, a significant increase of CS activity was observed in 3-AT treatment compared to CTR-HW (*p* < 0.05; Figure 5A). In addition, all supplemented feeds exhibited lower LDH activity levels compared to the CTR-HW treatment (*p* < 0.001; Figure 5B).

As for comparisons between day 53 and day 61 (for the same treatment), CS activity showed a significant decrease across all treatments (*p* < 0.05; Figure 5A) with the exception of 3-AT treatment. Particularly, the 1.5-AT treatment exhibited the most significant decrease, equivalent to a 53% reduction (*p* < 0.001; Figure 5A). No significant differences were observed in LDH activity (*p* > 0.05; Figure 5B).

### 3.4. Digestive Responses

**Figure 6:**
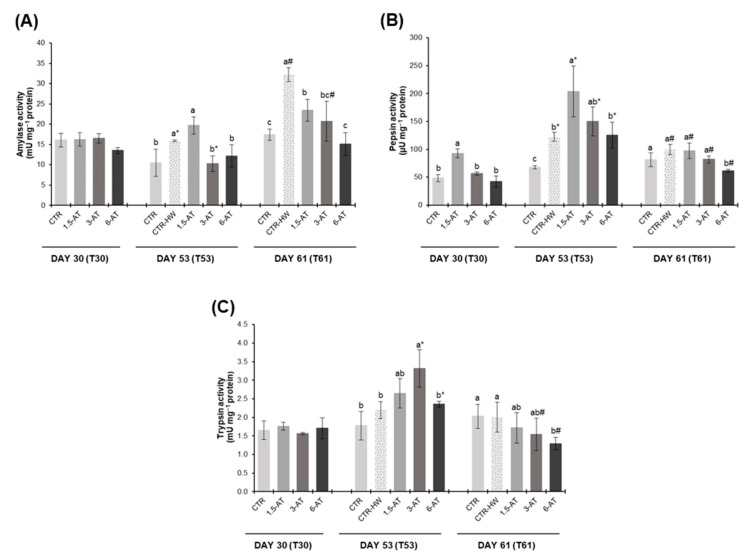
Digestive enzyme activities in the digestive tract of *D. sargus* fed the experimental diets at days 30 (T30, i.e., after 30 days of biofortification), 53 (T53, i.e., after 15 days of exposure to the MHW peak temperature), and 61 (T61, i.e., after 8 days of recovery from the MHW) of the trial (mean ± SD, *n* = 6). (**A**)—amylase activity (mU mg^−1^ protein); (**B**)—pepsin activity (µU mg^−1^ protein) and (**C**)—trypsin activity (mU mg^−1^ protein). Different letters denote significant differences between treatments on the same sampling day, and different symbols (* and #) indicate significant differences between sampling days T53 and T61 for the same treatment (*p* < 0.05). The absence of letters or symbols indicates no statistical difference. Abbreviations: CTR—control feed; CTR-HW—control feed exposed to the MHW; 1.5-AT—feed with 1.5% inclusion of *A. taxiformis*; 3-AT—feed with 3% inclusion of *A. taxiformis*; 6-AT—feed with 6% inclusion of *A. taxiformis*.

The activities of digestive enzymes (amylase, pepsin, and trypsin) in fish gut are presented in Figure 6. Thirty days of biofortification with *A. taxiformis* did not affect amylase and trypsin activities (*p* > 0.05; Figure 6A,C). However, fish fed with 1.5% macroalga revealed increased pepsin activity compared to non-biofortified ones (i.e., CTR treatment; *p* < 0.05; Figure 6B). At day 53 (MHW exposure), amylase and pepsin activities were significantly higher in the CTR-HW in relation to the CTR treatment (*p* < 0.05; Figure 6A,B). Amylase activity in CTR-HW treatment was also significantly higher than in fish fed with 3% and 6% *A. taxiformis* (*p* < 0.05; Figure 6A). On the other hand, fish from 1.5-AT treatment displayed increased pepsin activity in comparison with CTR-HW treatment (*p* < 0.001; Figure 6B), whereas significantly higher levels of trypsin activity (*p* < 0.05; Figure 6C) were found in 3-AT treatment. Upon recovery from the MHW (i.e., after 61 days), significantly higher levels of amylase activity were observed in the CTR-HW compared to CTR (*p* < 0.001), as well as to all biofortified treatments compared to CTR-HW (*p* < 0.05; Figure 6A). Regarding pepsin and trypsin activities, significant differences were only found between CTR-HW and 6-AT treatments (corresponding to a decrease of 38% and 36%, respectively; *p* < 0.05; Figure 6B,C).

Finally, comparing results of days 53 and 61 for the same treatment, a significant increase of amylase activity was spotted in CTR-HW and 3-AT treatments (*p* < 0.001; Figure 6A), while pepsin activity showed a significant decrease across all treatments after the MHW recovery period (*p* < 0.001; Figure 6B). Additionally, trypsin activity was significantly lower in the 3-AT and 6-AT treatments at day 61 (*p* < 0.001; Figure 6C).

### 3.5. Histopathology

**Figure 7:**
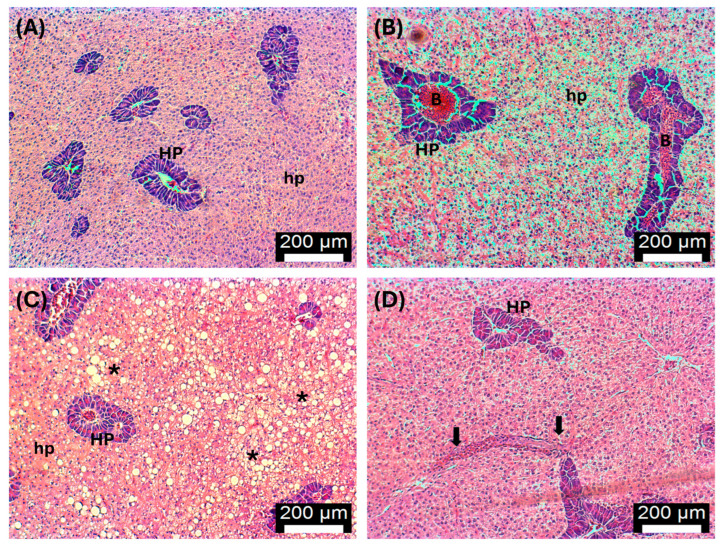
Histological changes in the liver of *D. sargus* exposed to a marine heatwave. (**A**) Control fish liver tissues at day 30 (T30, i.e., after 30 days of biofortification): normal hepatopancreas (HP) and hepatocytes (hp); (**B**) Liver exposed to 1.5% of *A. taxiformis* after biofortification (T30): normal hepatopancreas (HP) and hepatocytes (hp) with some increase of hepatopancreas volume and blood congestion (B); (**C**) Liver exposed to 1.5% of *A. taxiformis* after exposure to peak temperature of the MHW (T53): normal hepatopancreas (HP), hepatocytes (hp) and some fatty liver changes (steatosis) (*); (**D**) Liver exposed to 6% of *A. taxiformis* post-MHW (T61): liver and hepatopancreas (HP) showing some dilatation and blood congestion in the sinusoids (black arrows). Bar = 200 μm. H&E.

Most control fish livers, including those exposed to MHW (Figure 7A), showed a normal appearance with hepatocytes presenting the usual polygonal shape, located among the sinusoids and forming cord-like structures [54]. However, a few control fish showed signs of steatosis (Figure 7C). No consistent changes were detected in fish fed with 1.5-AT, although an apparent increase in hepatocyte volume (swelling) was observed in two fish (Figure 7B). Some fish fed with 3-AT and 6-AT revealed signs of hepatocyte swelling and bile stagnation, while others presented a normal structure (Figure 7D). Some fish fed with 6-AT also showed blood congestion and sinusoidal dilation (Figure 7D).

**Figure 8:**
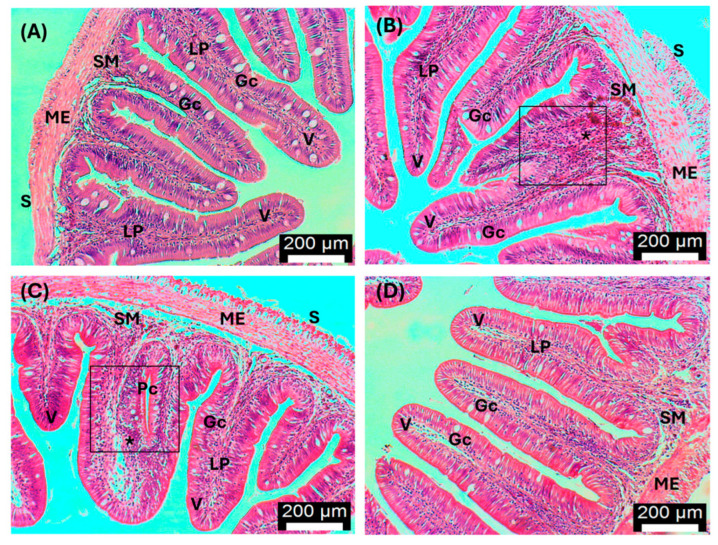
Histological changes in the intestines of *D. sargus* exposed to a marine heatwave. (**A**) Control fish intestine at day 30 (T30, i.e., after 30 days of biofortification): normal serosa (S), muscularis externa (ME), submucosa (SM), lamina propria (LP), villus (V), and goblet cells (Gc); (**B**) Liver exposed to 1.5% of *A. taxiformis* at day 30 (T30, i.e., after 30 days of biofortification): serosa (S), muscularis externa (ME), submucosa (SM), lamina propria (LP), villus (V), goblet cells (Gc), fusion of villi (square); cellular infiltration (*); (**C**) Liver exposed to 6% of *A. taxiformis* at day 30 (T30, i.e., after 30 days of biofortification): serosa (S), muscularis externa (ME), submucosa (SM), lamina propria (LP), villus (V), goblet cells (Gc), fusion of villi (square); cellular infiltration (*) and pseudocrypt (Pc); (**D**) Intestine exposed to 6% of *A. taxiformis*, post-MHW (T61): normal muscularis externa (ME), submucosa (SM), lamina propria (LP), villus (V), and goblet cells (Gc). Bar = 200 μm. H&E.

Regarding intestines (Figure 8), control fish and fish fed with the different concentrations of AT (1.5%, 3%, and 6%) presented a typical intestinal mucosal epithelium, which is composed of a single layer of high columnar epithelial cells and mucous cells.

## 4. Discussion

Extreme weather events prompted by climate change (e.g., MHWs) pose major challenges for the aquaculture and urge the development of efficient climate-smart strategies, to assure farmed seafood sustainability and security for future generations [8,55]. In this sense, the present study provides novel data on the potential use of seaweeds, particularly *A. taxiformis*, as a dietary-based eco-innovative approach to improve farmed marine fish performance and resilience in face of MHW events that are responsible for mass animal mortalities and, thus, substantial economic losses worldwide [2,11,13].

### 4.1. Biofortification with A. taxiformis

Overall, results showed that 30 days of biofortification with up to 6% of *A. taxiformis* did not compromise the welfare of *D. sargus* juveniles, nor hampered nutrient utilization (i.e., fitness, growth, and feed efficiency parameters were not negatively impacted by the seaweed supplementation), confirming that this seaweed may be safely used as an alternative functional ingredient in aquafeeds. This is also supported by the results of the histopathological analysis, where no consistent significant changes in the morphology of liver and intestinal tissues were found in animals fed with the different *A. taxiformis* concentrations tested (only minor alterations commonly observed in farmed animals were found, e.g., liver steatosis, and unlikely related to the presence of the macroalga). These results are consistent with the work of Thépot et al. [23], which found no adverse effects on the growth performance of Atlantic salmon (*Salmo salar*) fed diets containing *A. taxiformis* after four weeks. Instead, they observed an enhancement in growth rates with a 3% inclusion of whole dried *A. taxiformis*. Additionally, Peixoto et al. [29] reported that dietary supplementation with *Gracilaria* spp., a red macroalga, at inclusion levels of 2.5% or 7.5%, had no effect on growth performance and FCR of European seabass (*Dicentrarchus labrax*).

The fact that antioxidant enzymes activity (CAT and GST) and LPO levels in fish muscle, liver, and gut significantly decreased upon biofortification with the lowest levels of *A. taxiformis* inclusion (especially 1.5%) suggests a favorable maintenance of the redox state in these tissues or, in other words, a reduced requirement to scavenge reactive oxygen species (ROS) and prevent cellular damage. These results are in accordance with a previous study that reported decreased antioxidant enzymes activities, including SOD and glutathione peroxidase, as well as a reduction in lipid peroxidation products in the liver of rainbow trout (*Oncorhynchus mykiss*) upon supplementation with the red seaweed *Gracilaria pygmaea* [56]. Red macroalgae are a rich source of bioactive compounds, including sulphated polysaccharide such as carrageenan [57] and carotenoids like zeaxanthin, β-carotene, and fucoxanthin [58], all of which have demonstrated antioxidant and free radical scavenging properties [56]. In this context, it is plausible that upon incorporating *A. taxiformis* in aquafeeds, the bioactive compounds present in the macroalga have contributed to enhance the antioxidant capacity of biofortified aquafeeds, which improved fish tissues’ ability to scavenge free radicals and prevent lipid peroxidation products. However, it is worth mentioning that the positive outcomes of *A. taxiformis* supplementation were dose- and tissue-dependent, as concentrations of *A. taxiformis* above 3% led to increased oxidative stress in fish gut. Given that the gastrointestinal tract is the first organ directly exposed to nutrients and bioactive compounds released from feeds during digestion, it is particularly sensitive to changes in dietary patterns [59]. The intestine is known for its rapid cell turnover, making it more prone to oxidative stress, which, in turn, calls for enhanced antioxidant scavengers’ activity [59,60]. Furthermore, these findings are aligned with the study of Marmelo et al. [46], which revealed a dose-dependent relationship in the antioxidant response of juvenile *Sparus aurata* biofortified with *Laminaria digitata*, a brown macroalga, with the most favorable outcomes observed at the lowest inclusion percentages (1.5%).

The determination of CS and LDH activities constitutes a proxy to understand the extent to which aerobic and anaerobic pathways are being favored in individuals. The absence of statistical differences upon 30 days of biofortification at optimal rearing conditions (i.e., before the simulation of a MHW event) indicates that the supplementation with *A. taxiformis* in doses up to 6% did not imply an increased energetic expenditure in nutrient conversion nor hampered fish metabolism, a result that was further corroborated by the absence of significant effects in fish fitness and growth parameters.

Intestinal enzymes play a crucial role in fish nutritional status, and the activities of pancreatic secretion enzymes, like trypsin and amylase, are often used as indicators of the digestive capacity of farmed fish [61,62]. The absence of significant differences in trypsin and amylase activities of non-biofortified and biofortified *D. sargus* indicates that *A. taxiformis* did not affect fish digestive capacity. Pepsin is also recognized as an essential digestive enzyme, playing a significant role in protein digestion in fish feeds [63]. Hence, the significant increase in pepsin activity observed in the fish supplemented with 1.5% of *A. taxiformis* suggests that the lower inclusion level stimulated the secretion of this enzyme, potentially improving protein digestion.

### 4.2. Interactive Effects of MHW and A. taxiformis Biofortification

In general, organisms exposed to temperatures outside their thermal plasticity range tend to exhibit a fitness decline due to the additional energetic costs and/or need to redirect energy (that could be used for somatic growth under optimal environmental conditions) towards the maintenance of internal homeostasis [64,65,66]. Yet, in the present study, juvenile *D. sargus* fitness indicators were not significantly affected by the exposure to a category II MHW (i.e., at day 53), suggesting that the conditions during the simulation of this extreme event were either still within the thresholds of *D. sargus* thermal plasticity, or not extended in time long enough to cause irreversible damage at the whole organism’s level. However, the fact that non-biofortified fish exhibited a significant WG reduction upon 8 days of recovery from MHW (at day 61) suggests that fish physiological mechanisms may have become less effective over time and, thus, the thermal stress experienced during the MHW had somewhat cumulative effects. The similar principle could also be extended to fish fed with 6% *A. taxiformis*, which also exhibited a reduction in WG upon 61 days of trial (i.e., after recovering from the MHW). This decrease in WG, evident only in fish exposed to the highest inclusion level, might result from the presence of bioactive compounds in *A. taxiformis* that can, beyond a certain concentration and feeding duration, lose their beneficial attributes [67]. Seaweeds contain a wide array of bioactive compounds such as phenolics, whose incorporation in diets have demonstrated conflicting effects on growth performance. In fact, high doses of polyphenols from macroalgae have shown anti-nutritional effects, impacting both growth performance and nutrient utilization efficiency [68]. All in all, this finding suggests that the use of higher doses of *A. taxiformis* (>3%) for extended periods should be avoided to ensure maximum animal performance.

Fish are particularly susceptible to thermal stress-induced oxidative damage, as elevated temperatures lead to increased ROS production [69,70]. To mitigate the effects of thermal stress, fish activate their enzymatic antioxidant defense mechanisms [71,72]. However, if these defenses are inadequate and/or stress becomes too severe or long-lasting, cell damage can take place giving rise to increased LPO levels [11]. In this study, exposure to a MHW enhanced CAT and GST activities in the muscle, gut, and liver of non-biofortified fish, compared to those reared under optimal temperature conditions. Despite the activation of these antioxidant scavengers, ROS formation was not fully prevented, culminating in increased LPO levels in the tissues of non-biofortified fish exposed to the MHW. In accordance with these results, previous studies on marine organisms have reported increased CAT, GST, and SOD activities, as well as elevated LPO levels under higher temperatures [11,12]. Concerning *A. taxiformis* potential to prevent the damages elicited by MHWs, a significant decrease in CAT and GST activities, as well as LPO levels, was observed in the muscle, gut, and liver tissues, particularly in fish biofortified with the lowest inclusion level (1.5%), indicating considerable beneficial outcomes of this macroalga at lower doses. Concurrently, GST activity was induced in the gut and liver tissues of fish biofortified with 3% of *A. taxiformis*. This result is suggestive of an enhanced metabolization of undesired substances (e.g., ROS or even a somewhat toxic compound), as GST is particularly involved in the biotransformation of non-essential and/or potentially toxic compounds. Yet, further research would be necessary to elucidate the specific mechanisms involved, as there is no information on the levels of potentially bioactive harmful compounds in the experimental feeds used in this trial. Additionally, the influence of *A. taxiformis* as a functional ingredient on the antioxidant responses of *D. sargus* when coping with high temperatures has not been reported yet, hampering comparison with other studies. Nevertheless, it should be emphasized that fish biofortified with *A. taxiformis* evidenced significantly lower LPO levels, regardless of seaweed dose, and this result, by itself, constitutes a clear benefit to animals exposed to acute and severely stressful rearing conditions.

Upon 8 days of recovery from the MHW (day 61), LPO levels significantly decreased in all treatments and tissues in relation to values observed during the MHW (day 53; except for the liver, where only the CTR-HW showed a significant decrease), with the most substantial reduction observed in fish fed 3% and 1.5% of *A. taxiformis* in muscle and gut, respectively. This result is aligned with the overall trend of decreased activity of antioxidant enzymes in the different tissues. This decline could be attributed to a restoration of redox balance and a decrease in oxidative stress due to the cessation of the MHW stressor, consequently leading to a reduced demand for antioxidant enzyme activity, as the requirement to counteract ROS diminishes. Additionally, it is worth mentioning that concentrations of *A. taxiformis* above 3% showed the greatest reductions in CAT and GST activity levels. The recovery effects were more pronounced in fish fed diets containing 3% and 6% of *A. taxiformis*, probably due to their initially higher activity levels on day 53, which declined after 8 days from the MHW cessation.

Warming is one of the most critical environmental threats to marine ectotherms, particularly due to its impact on metabolic processes [47]. Thermal stress has been reported to cause drastic changes in animals’ metabolic performance, mostly related to the need to adapt and reallocate energy [73,74], ultimately resulting in a shift in the energy production process [47]. After exposure to the MHW, a significant increase in LDH activity was observed in the muscle of non-biofortified fish. Such enhancement of the anaerobic pathway, over aerobic ones, may be linked to the pronounced oxidative stress response (e.g., induced CAT activity and LPO levels in the muscle tissue) prompted by thermal stress in non-biofortified fish. Under stressful conditions, such as exposure to high temperatures, the anaerobic pathway becomes activated, resulting in an increased concentration of lactate in tissues and higher LDH activity [75,76]. However, this strategy is not viable in the long term due to finite stores of fermentable substrate and cytotoxicity, especially considering the anticipated chronic effects of climate change in the future [47]. Interestingly, fish biofortified with 1.5% *A. taxiformis* showed a significant increase in CS activity, paralleled by a concomitant decrease in LDH activity, suggesting an enhancement of the aerobic capacity potential. The decrease in LDH further supports this trend, as it implies a reduced reliance on anaerobic metabolism. The incorporation of 6% *A. taxiformis* also led to a reduction in LDH activity; however, this decrease was less pronounced compared to the lowest inclusion level, indicating that increasing the macroalga concentration does not confer any additional metabolic benefit. Altogether, the present findings showed that the inclusion of *A. taxiformis* (particularly at inclusion levels below 3%) constitutes an asset from the fish metabolic standpoint.

During the recovery period, CS activity decreased in all treatments, except in fish biofortified with 3% of *A. taxiformis*, most likely due to exhaustion and energy deprivation motivated by the need to perform rapid metabolic/energetic adjustments during the ramp temperature increase and decrease of the MHW. Notably, the most significant decrease in CS activity was observed in fish biofortified with 1.5% *A. taxiformis*, suggesting that the positive outcomes promoted by the macroalga at the lowest inclusion level may be time-limited.

Digestive processes and nutrient digestibility typically decline at temperatures beyond the optimal range, mainly due to the influence of temperature on digestive enzyme activities [77]. In addition, dietary ingredients have been reported to also affect digestive enzyme activity in several fish species. Non-biofortified *D. sargus* revealed a significant increase in amylase activity after exposure to the MHW. These results are consistent with those of Pereira et al. [78], who reported a significant increase in amylase activity in *D. labrax* with increasing temperature. The influence of temperature on amylase activity has been observed in both freshwater and marine fish [79], where higher temperatures improve carbohydrate digestibility up to a physiologically appropriate threshold for the species [78]. Conversely, fish fed with 3% and 6% of *A. taxiformis* showed significantly lower amylase activity. In plants, soluble non-starch polysaccharide (NSP) forms functional networks that bind to water or minerals, exchange cations, and adsorb organic compounds [80]. Seaweeds’ soluble NSPs, which contribute to increased viscosity of the diet and the intestinal digesta [81], could be a possible explanation for the decrease of amylase activity levels. The NSPs are viscous in nature, thereby decreasing the rate of diffusion of digestive enzymes to substrates, hindering the effective interaction between substrate and digestive enzymes at the mucosal surface [82,83]. This can occur throughout the entire gastrointestinal tract of fish and may be especially significant in the gut of juvenile fish [80]. Another possible cause could be attributed to the fact that amylase activity can be negatively influenced by the dietary starch content [84]. The results obtained in this study align with the previous hypothesis, as the starch content in the experimental diets decreased with the increasing percentages of *A. taxiformis* inclusion (from 13.7% to 9.9% due to alterations in wheat meal content; see Table 1), resulting in the reduction of amylase activity observed in fish fed diets with higher macroalga concentrations (i.e., 3% and 6% inclusion levels). Similarly to amylase, an enhancement of pepsin activity was observed in fish from the control treatment after exposure to the MHW. Mazumder et al. [63] also observed a significant increase in pepsin activity in *Lutjanus malabaricus* subjected to increasing temperatures between 26 °C and 30 °C. Additionally, fish biofortified with 1.5% *A. taxiformis* showed significantly higher pepsin activity levels, further supporting the efficiency of the lowest inclusion level in improving protein digestion even under thermally stressful conditions. Lastly, the increased temperatures experienced during the MHW did not affect trypsin activity of non-biofortified fish. On the other hand, a significant increase in trypsin activity was observed in fish fed 3% of *A. taxiformis*, suggesting a beneficial impact of the seaweed on the hydrolysis of dietary proteins.

As for the recovery period (day 61), biofortification with 3% *A. taxiformis* increased amylase activity (in relation to day 53), while pepsin activity levels significantly decreased in all treatments, both non-biofortified and biofortified. This decline can be likely attributed to the temperature decrease experienced between days 53 and 61, as increased pepsin activities are usually associated with higher temperatures. Among biofortified treatments, fish fed with 1.5% and 3% *A. taxiformis* revealed the most pronounced decreases in pepsin and trypsin activities between day 53 and day 61, respectively, which could be attributed to their initially higher activity levels observed on day 53. Nevertheless, despite the changes elicited by MHW and biofortification on digestive enzyme activities, nutrient conversion and the overall growth performance of fish remained consistent throughout time.

## 5. Conclusions

The present study highlights the potential of *A. taxiformis* as a promising alternative functional feed ingredient that can be used in a climate-smart perspective, with the purpose of improving juvenile marine fish antioxidant, metabolic, and digestive responses to adverse environmental conditions, such as MHW events. Overall, lower inclusion percentages (particularly 1.5%) seemed to be more effective in counteracting the thermal stress elicited by MHW. This resilience was evidenced by the decrease in tissues’ oxidative stress, most likely related to the presence of antioxidant bioactive compounds in *A. taxiformis*, which promoted an enhancement of fish tissues’ antioxidant capacity and ability to prevent cellular damage. Additionally, lower doses of *A. taxiformis* improved aerobic potential and reduced dependence on less efficient anaerobic pathways during temperature stress, as well as improved the digestion efficiency of aquafeeds. Considering the less favorable physiological outcomes observed in fish biofortified with higher seaweed inclusion percentages, together with the higher costs implied, future studies in this direction should prioritize supplementation doses below 3%.

As a final remark, it should be emphasized that the present findings are limited to the severity and duration of the thermal stress simulated in this experimental design. The conditions used in this study were selected to reflect specific scenarios representative of real marine events in coastal and shallow water zones in this region. However, MHW conditions can vary significantly across different regions of the globe. Therefore, it becomes essential for future research to evaluate the consistency of the findings obtained in this study by investigating a wider array of thermal stress conditions, enabling a deeper interpretation of the potential impacts of incorporating *A. taxiformis* in aquafeeds on the physiology of the species studied.

## Figures and Tables

**Table 1:** Proximate ingredient composition of CTR and AT-enriched feeds (% dry matter). Abbreviations: CTR—control feed; 1.5-AT—1.5% inclusion of *A. taxiformis*; 3-AT—3% inclusion of *A. taxiformis*; 6-AT—6% inclusion of *A. taxiformis*.

	Diets			
Ingredients (%)	CTR	1.5-AT	3-AT	6-AT
Fishmeal Super Prime ^a^	25.0	25.0	25.0	25.0
Fish protein concentrate ^b^	2.0	2.0	2.0	2.0
Soy protein concentrate ^c^	10.0	10.0	10.0	10.0
Pea protein concentrate ^d^	3.0	3.0	3.0	3.0
Wheat gluten ^e^	6.5	6.5	6.5	6.5
Corn gluten meal ^f^	10.0	10.0	10.0	10.0
Soybean meal 44 ^g^	6.0	6.0	6.0	6.0
Rapeseed meal ^h^	6.0	6.0	6.0	6.0
Wheat meal ^i^	10.8	9.3	7.8	4.8
Faba beans (low tannins) ^j^	6.0	6.0	6.0	6.0
Vitamin and mineral premix ^k^	1.0	1.0	1.0	1.0
Choline chloride 50% ^l^	0.2	0.2	0.2	0.2
Monoammonium phosphate ^m^	1.2	1.2	1.2	1.2
Fish oil ^n^	5.0	5.0	5.0	5.0
Soybean oil ^o^	7.3	7.3	7.3	7.3
*Asparagopsis taxiformis* ^p^	-	1.5	3.0	6.0
**Proximate composition (%)**				
Dry matter, DM (%)	94.2	94.0	93.9	94.1
Crude protein	46.0	46.0	45.9	45.7
Crude fat	16.0	16.0	16.1	16.1
Fiber	1.8	1.9	2.0	2.1
Starch	13.7	12.8	11.8	9.9
Ash	6.8	7.1	7.4	8.0
Gross energy (MJ/kg feed)	21.0	21.0	20.9	20.8

^a^ Diamante, Pesquera Diamante, Peru (crude protein, CP: 66.3% dry matter, DM; crude fat, CF: 11.5% DM). ^b^ CPSP90, Sopropêche, France (CP: 82.6% DM; CF: 9.6% DM). ^c^ Soycomil P, ADM, The Netherlands (CP: 62.2% DM; CF: 0.7% DM). ^d^ Lysamine GPS, Roquette, France (CP: 78.1% DM; CF: 8.3% DM). ^e^ VITAL, Roquette, France (CP: 80.4% DM; CF: 5.8% DM). ^f^ COPAM, Portugal (CP: 61.2% DM; CF: 5.2% DM). ^g^ Solvent extracted, Ribeiro and Sousa Lda, Portugal (CP: 43.8% DM; CF: 3.5% DM). ^h^ Solvent extracted, Ribeiro and Sousa Lda, Portugal (CP: 34.3% DM; CF: 2.1% DM). ^I^ Molisur, Spain (CP: 11.7% DM; CF: 1.6% DM). ^j^ Ribeiro and Sousa, Portugal (CP: 24.5% DM; CF: 1.7% DM). ^k^ Premix for marine fish, PREMIX Lda, Portugal. Vitamins (IU or mg kg^−1^ diet): DL-alpha-tocopherol acetate, 100 mg; sodium menadione bisulphate, 25 mg; retinyl acetate, 20,000 IU; DL-cholecalciferol, 2000 IU; thiamine, 30 mg; riboflavin, 30 mg; pyridoxine, 20 mg; cyanocobalamin, 0.1 mg; nicotidin acid, 200 mg; folic acid, 15 mg; ascorbic acid, 1000 mg; inositol, 500 mg; biotin, 3 mg; calcium panthotenate, 100 mg; choline chloride, 1000 mg, betaine, 500 mg. Minerals (g or mg kg^−1^ diet): cobalt carbonate, 0.65 mg; copper sulphate, 9 mg; ferric sulphate, 6 mg; potassium iodide, 0.5 mg; manganese oxide, 9.6 mg; sodium selenite, 0.01 mg; zinc sulphate. 7.5 mg; sodium chloride, 400 mg; calcium carbonate, 1.86 g; excipient wheat middling’s. ^l^ ORFA, The Netherlands. ^m^ Windmill AQUAPHOS, ALIPHOS, The Netherlands. ^n^ Sopropêche, France, 16% EPA, 12% DHA (CF: 98.1% DM). ^o^ JC Coimbra, Portugal (CF: 98.6% DM). ^p^ SeaExpert, Fail Island, Azores, Portugal.

**Table 2:** Weight (W), total length (TL), Fulton’s condition index (K), hepatosomatic index (HSI), weight gain (WG), specific growth rate (SGR) and feed conversion ratio (FCR) of juvenile *D. sargus* after 30 (T30, i.e., after 30 days of biofortification), 53 (T53, i.e., after 15 days of exposure to the MHW peak temperature) and 61 (T61, i.e., after 8 days of recovery from the MHW) days of trial (mean ± SD, *n* = 12).

		W (g)	TL (cm)	K	HSI (%)	WG (%)	SGR (% day^−1^)	FCR
**T30**	**CTR**	48.98 ± 7.41	13.95 ± 0.75	1.80 ± 0.23	0.67 ± 0.10	83.36 ± 16.17	1.71 ± 0.25	0.74 ± 0.16
	**1.5-AT**	52.23 ± 9.18	14.28 ± 0.78	1.79 ± 0.23	0.59 ± 0.05	92.06 ± 26.74	2.37 ± 0.36	0.69 ± 0.18
	**3-AT**	56.47 ± 11.32	14.38 ± 0.86	1.88 ± 0.09	0.60 ± 0.10	83.66 ± 19.90	1.78 ± 0.35	0.75 ± 0.18
	**6-AT**	50.31 ± 8.11	14.28 ± 0.90	1.73 ± 0.24	0.58 ± 0.14	66.86 ± 17.63	1.70 ± 0.26	0.95 ± 0.23
**T53**	**CTR**	64.78 ± 10.29	15.18 ± 0.99	1.85 ± 0.20	0.70 ± 0.18	138.63 ± 32.83	1.56 ± 0.31	0.78 ± 0.18
	**CTR-HW**	56.55 ± 10.49	14.55 ± 0.82	1.82 ± 0.06	0.50 ± 0.08	107.42 ± 32.83	1.29 ± 0.31	1.05 ± 0.35
	**1.5-AT**	53.49 ± 9.12	14.47 ± 0.95	1.76 ± 0.18	0.54 ± 0.09	96.71 ± 25.87	1.19 ± 0.33	1.14 ± 0.30
	**3-AT**	57.41 ± 7.49	15.03 ± 0.74	1.69 ± 0.10	0.52 ± 0.09	94.36 ± 22.12	1.33 ± 0.25	1.15 ± 0.24
	**6-AT**	55.95 ± 9.71	14.92 ± 0.71	1.67 ± 0.12	0.49 ± 0.07	84.19 ± 18.58	1.27 ± 0.32	1.29 ± 0.29
**T61**	**CTR**	65.28 ± 13.17	15.22 ± 1.13	1.84 ± 0.16 ^a^	0.54 ± 0.25	155.31 ± 20.12 ^a^	1.37 ± 0.38	0.77 ± 0.10
	**CTR-HW**	56.01 ± 9.82	14.83 ± 0.79	1.70 ± 0.15 ^ab^	0.77 ± 0.19	109.33 ± 15.96 ^b^	1.12 ± 0.34	1.10 ± 0.16
	**1.5-AT**	58.86 ± 6.96	15.15 ± 0.55	1.69 ± 0.15 ^ab^	0.48 ± 0.14	111.81 ± 23.15 ^bc^	1.22 ± 0.20	1.22 ± 0.25
	**3-AT**	56.03 ± 5.79	14.82 ± 0.64	1.72 ± 0.05 ^ab^	0.64 ± 0.13	102.96 ± 14.63 ^bc^	1.14 ± 0.18	1.22 ± 0.12
	**6-AT**	54.69 ± 11.60	14.90 ± 0.99	1.64 ± 0.09 ^b^	0.72 ± 0.29	76.22 ± 15.16 ^c^	1.08 ± 0.33	1.33 ± 0.42

In each column, different letters denote significant differences between treatments on the same sampling day. The absence of letters indicates no statistical difference. Abbreviations: CTR—control feed; CTR-HW—control feed exposed to the MHW; 1.5-AT—feed with 1.5% inclusion of *A. taxiformis*; 3-AT—feed with 3% inclusion of *A. taxiformis*; 6-AT—feed with 6% inclusion of *A. taxiformis*.

## Data Availability

Data is contained within the article.
